# Transcriptome analysis of response to *Plasmodiophora brassicae* infection in the Arabidopsis shoot and root

**DOI:** 10.1186/s12864-017-4426-7

**Published:** 2018-01-05

**Authors:** Solmaz Irani, Brett Trost, Matthew Waldner, Naghabushana Nayidu, Jiangying Tu, Anthony J. Kusalik, Christopher D. Todd, Yangdou Wei, Peta C. Bonham-Smith

**Affiliations:** 1 Department of Biology, University of Saskatchewan, Saskatoon, S7N 5E2 Canada; 2 Department of Computer Science, University of Saskatchewan, Saskatoon, S7N 5C9 Canada

**Keywords:** *Arabidopsis thaliana*, Clubroot, Host-pathogen interaction, *Plasmodiophora brassicae*, RNA-seq, Root, Shoot, Transcriptomic profile

## Abstract

**Background:**

Clubroot is an important disease caused by the obligate parasite *Plasmodiophora brassicae* that infects the Brassicaceae. As a soil-borne pathogen, *P. brassicae* induces the generation of abnormal tissue in the root, resulting in the formation of galls. Root infection negatively affects the uptake of water and nutrients in host plants, severely reducing their growth and productivity. Many studies have emphasized the molecular and physiological effects of the clubroot disease on root tissues. The aim of the present study is to better understand the effect of *P. brassicae* on the transcriptome of both shoot and root tissues of *Arabidopsis thaliana*.

**Results:**

Transcriptome profiling using RNA-seq was performed on both shoot and root tissues at 17, 20 and 24 days post inoculation (dpi) of *A. thaliana*, a model plant host for *P. brassicae*. The number of differentially expressed genes (DEGs) between infected and uninfected samples was larger in shoot than in root. In both shoot and root, more genes were differentially regulated at 24 dpi than the two earlier time points. Genes that were highly regulated in response to infection in both shoot and root primarily were involved in the metabolism of cell wall compounds, lipids, and shikimate pathway metabolites. Among hormone-related pathways, several jasmonic acid biosynthesis genes were upregulated in both shoot and root tissue. Genes encoding enzymes involved in cell wall modification, biosynthesis of sucrose and starch, and several classes of transcription factors were generally differently regulated in shoot and root.

**Conclusions:**

These results highlight the similarities and differences in the transcriptomic response of above- and below-ground tissues of the model host Arabidopsis following *P. brassicae* infection. The main transcriptomic changes in root metabolism during clubroot disease progression were identified. An overview of DEGs in the shoot underlined the physiological changes in above-ground tissues following pathogen establishment and disease progression. This study provides insights into host tissue-specific molecular responses to clubroot development and may have applications in the development of clubroot markers for more effective breeding strategies.

**Electronic supplementary material:**

The online version of this article (10.1186/s12864-017-4426-7) contains supplementary material, which is available to authorized users.

## Background

Clubroot, caused by the soil-borne obligate plant pathogen *Plasmodiophora brassicae,* is a serious disease of Brassica crops, such as the important oilseed canola, resulting in decreased yields in Canada and worldwide [1]. The disease significantly affects canola seed quality by reducing oil content and seed weight [2]. *P. brassicae* is classified as a protist belonging to the subgroup Rhizaria [3]. The life cycle of the pathogen is comprised of a primary phase when resting spores in the soil germinate and penetrate a host root hair in the form of zoospores as soon as an appropriate host plant is available. During the second phase of the life cycle, secondary plasmodia form in the root cortex leading to the production of visible galls. The subsequent formation of root galls results in reduced uptake of water and nutrients by the plant, wilting, reduced plant growth, and a reduced seed set [4]. Mature secondary plasmodia eventually form resting spores that are released into the soil, where they are long-lived and resistant to severe environmental conditions, making it impossible to prevent the disease by chemical treatments or crop rotation [5]. Therefore breeding, together with resistant crop varieties, will be the most efficient tools to control clubroot disease compared to other agricultural control strategies [6]. An understanding of the molecular basis of the host response to pathogen infection will guide the development of these new tools.

A previous proteomic study of Arabidopsis root and hypocotyls revealed that *P. brassicae* infection altered the abundance of 12% of proteins, mainly related to cell defence, differentiation, and general metabolism when compared to uninfected samples [7]. In *Brassica napus,* significant changes in several proteins involved in hormone metabolism, cell wall metabolism, and detoxification were observed in root during clubroot establishment [8]. A number of genomic studies have attempted to identify the host defence response during *P. brassicae* infection and clubroot progression. More than 1000 differentially expressed genes (DEGs) were reported in clubroot-infected Arabidopsis root compared with control root [9]. The DEGs included genes for growth and cell cycle control, as well as starch, lipid, sugar, and flavonoid metabolism [9]. Microarray analysis of gene expression during the primary phase of the *P. brassicae* lifecycle in Arabidopsis root showed that the expression of several genes related to signal transduction, primary and secondary metabolisms, and cell wall modification was altered [10]. It was suggested that during early pathogen establishment, salicylic acid (SA) and ethylene (ET) biosynthesis genes were downregulated and jasmonic acid (JA) biosynthesis genes were upregulated [10]. Susceptibility or resistance to *P. brassicae* infection and subsequent clubroot establishment are thought to be a result of the host’s ability to regulate primary metabolism, transcription factor activities (TFs), defence responses, and cell division [11]. WRKY-type TFs were found to be upregulated by pathogen infection while other TF families DEGs were downregulated [11]. Microarray analysis of Arabidopsis root transcriptional changes during distinct developmental stages of *P. brassicae* revealed the upregulation of auxin, cytokinin and brassinosteroid metabolism and signaling genes [12]. The clubroot resistance gene, *Rcr1*, has been functionally characterised in *Brassica rapa*, and in plants carrying *Rcr1* the biosynthesis of JA, ET, and indole-containing compounds, defensive callose deposition, and expression of TFs were upregulated by infection [13]. Differentially expressed TF families primarily included WRKY, MYB, BHLH, AP2/ERF and ET-responsive families [13]. Between resistant and susceptible *B. rapa* lines, genes associated with Ca^2+^ influx, hormone signaling, TFs, cell wall modification, cell division and expansion was differentially regulated [14]. Similarly, several genes encoding NBS-LRR proteins and chitinases, and genes involved in SA, and Ca^2+^ signaling pathways, biosynthesis of cell wall components and glucosinolates were upregulated in root tissue of clubroot-resistant wild cabbage when compared to clubroot-susceptible broccoli following *P. brassicae* infection [15]. Transcriptome analysis of Arabidopsis infected root at 1 and 2 days after inoculation showed that early stage of infection regulates the expression of several flavonoid, lignin, auxin, cytokinin and receptor-kinase genes [16].

Genome analyses of *P. brassicae* has identified an absence of genes encoding sulfur and nitrogen uptake proteins as well as the synthesis of 'several' metabolites such as histidine, tryptophan and thiamine suggesting, that *P. brassicae* relies on the host for these metabolites [5, 17]. Furthermore, the small size of the *P. brassicae* genome, compared to other obligate biotrophic pathogens [17], suggests that it depends on the host plant for carbon sources for its growth. The pathogen induces the upregulation of host auxin (indole-3-acetic acid, IAA), JA, and cytokinin (CK) pathways, and downregulation of the SA pathway during early infection [5, 17].

Previous studies on *P. brassicae* infection have focused on clubroot development or pathogen life cycle in the host root. We are not aware of any studies that have compared the impact of *P. brassicae* infection on the above- and below-ground transcriptomic responses of infected plants. In this study, we describe the transcriptomic changes in the shoot and root of Arabidopsis during clubroot disease establishment and progression. Our results provide new insight into the whole-plant molecular response to *P. brassicae* infection and clubroot progression as well as providing above ground indicators/markers for below-ground infection.

## Methods

### Biological materials and pathogen inoculation

*Arabidopsis thaliana* ecotype Columbia (Col.0, fully susceptible to clubroot disease) seed and *Plasmodiophora brassicae* pathotype P3 (Pb3) isolate were used in this study. Canola (*Brassica napus*) plants, cultivar Westar, were inoculated with *P. brassicae* resting spores at rosette stage (fourth leaf stage). Fresh galls with *P. brassicae* resting spores were collected from infected roots when plants were flowering. Galls were ground in a sterilized mortar using a pestle to extract resting spores and further homogenized in deionised water (1w:3v) before filtering through cheesecloth (Grade 5). Spore density was determined using a haemocytometer.

Arabidopsis seeds were germinated in peat mix soil in a growth chamber (Conviron) under 16/8 h light/dark cycle, 100 μmol (photons) m^−2^ s^−1^ and a constant 22 °C. Ten-day old uniform seedlings were transferred to Sunshine Mix #3 soil (Sun Gro Horticulture Inc., Vancouver, BC) with four plants in each square plastic 10 cm pot (350 ml volume). Seedlings were allowed to acclimatise in these pots for three additional days before a resting spore suspension (400 μL of 5 × 10^7^ resting spores/mL) was added to the surface of the soil immediately around each plant. Control plants, inoculated with 400 μL of distilled water, were grown in separate trays in the same growth chamber. For each time period, 3 to 6 pots (12–24 plants) were sampled. Plants were treated in blocks, sampled randomly and the experiment was replicated three times. Pots were watered as required and 300 mg L^−1^ of 20–20-20 Plant-Prod® fertilizer (Master Plant-Prod Inc.) was added to the water once a week.

### Disease assessment and microscopic analysis

The disease index (DI) of infected plants at the three time points, 17, 20 and 24 days post inoculation (dpi) was determined according to Siemens et al. [18]. Root infection was assessed on a 0–4 scale, 0, no symptom; 1, very small galls mainly on lateral roots; 2, small galls on the main and lateral roots; 3, medium-sized galls with possible negative effect on plant growth; and 4, severe galls on both main and lateral roots, deformed roots, and impaired growth. Three independent replicates of 20 plants were used to measure the disease index of plants.

For microscopic analysis, infected Arabidopsis roots were sampled for cortical infection observation at 17, 20 and 24 dpi. Tissues were fixed overnight in a freshly prepared solution of 2% glutaraldehyde in 0.1 M phosphate buffer (PB) pH 7.4 at room temperature, washed three times in fresh PB for 30 min and post-fixed in 1% osmium tetroxide (EMS; VWR, Canada) prepared in 0.1 M PB for 4 h and then washed three times in distilled water for 30 min each wash. Tissues were dehydrated through a grade series of ethanol before incubation in propylene oxide (Alfa Aesar; VWR, Canada) for 8 h followed by embedding in a mold in 100% resin and incubated at 60 °C for polymerization. Ultra-thin sections (60 nm) were generated with a Microstar diamond knife (Huntsville, US) on a Reichert-Jung microtome (Reichert microscopic service; Depew, US) and captured onto single slot copper grids coated with Formvar or 100 naked mesh copper grids. Sections were post-stained with 2% uranyl acetate (EMS; VWR, Canada) for 30 min in the dark, followed by Reynold’s lead citrate solution for 10 min [19], and observed with a Hitachi HT7700 transmission electron microscope (TEM).

### RNA isolation, RNA-seq library preparation, and gene expression analysis

Infected shoot and root at 17, 20 and 24 dpi, and non-infected samples at the same time points were collected for RNA-seq analysis. The TruSeq RNA sample preparation kit (Illumina, San Diego, CA, USA) was used for RNA extraction and library construction, according to the manufacturer’s protocol. Library sequencing (100 cycles) was conducted from both ends on an Illumina HiSeq 2500 (NRC, Saskatoon). The reads were trimmed using trimmomatic ver.0.30 [20] with minimum quality score 15, removing the first 12 bp, and then aligned to the *A. thaliana* genome using TopHat ver. 2.0.7 [21]; non-default parameters were minimum intron length 20, maximum intron length 11,000, and mean distance between paired ends-reads 30. For genome and pathway annotations, ver. 10 of the Arabidopsis Information Resource (TAIR) annotation dataset was used [22]. In order to assign biological process to the DEGs, GO (Gene Ontology) enrichment analysis was carried out using the Gene Ontology tool (http://geneontology.org) [23, 24]. The GO tool on http://geneontology.org/ is the PANTHER GO tool located at http://pantherdb.org. The DEGs with log_2_ fold change ≥1 and *P* ≤ 0.05 were selected for GO enrichment analysis. The percentage of mapped genes to each Go term was calculated by dividing the number of mapped genes to the specific biological process by the number of all selected genes. Cufflinks was used to generate Fragments Per Kilobase of transcript per Million mapped reads (FPKM) values for each gene [25]. Differential expression analysis was performed using DeSeq2 [26] following the recommended RNA-seq workflow [27]. MapMan software was used to analyse differential expression in metabolic pathways [28]. Heat maps were generated using the gplots package in R [29].

### Validation of RNA-seq data by qPCR

To confirm the RNA-seq results, qPCR was performed on a selection of 15 genes identified in the shoot data and 11 genes identified in the root data. RNA was extracted from samples of infected and control tissues, at 17, 20, and 24 dpi, using an E.Z.N.A Plant RNA Kit (Omega Bio-Tek, Norcross, GA, USA). RNA concentration was quantified by NanoDrop 2000 spectrophotometer (ThermoFisher Scientific) and 2 μg of total RNA was used to synthesize cDNA using the QuantiTect® Reverse Transcription Kit (Qiagen, Germany) according to the manufacturer’s protocol. qPCRs were conducted with an iCycler iQ5 Multicolor real time PCR detection system (Bio-Rad) using the Platinum® SYBR® Green qPCR SuperMix-UDG kit (Invitrogen). A final volume of 25 μL was used for the reactions, with 12.5 μL of SYBR Green mix, 0.25 μM of each primer and final concentration of 2 mM MgCl_2_. Thermal cycling conditions were 95 °C for 4 min, 40 cycles of 95 °C for 20 s, followed by 57–65 °C (depending on the primer-set used, Additional file 1) for 20 s, and 72 °C for 30 s. PCR amplification efficiency rate was tested for all primers by the standard curve method [30]. The transcript level of genes was normalized using two reference genes *ACTIN2* (At3g18780) and *UBQ10* (At4g05320) [31]. The relative transcript abundance of control (uninfected) samples at each time point was set to 1, to which the infected sample at the same time point was compared. Relative expression of genes was calculated using the 2^-ΔΔCt^ method [32]. The two reference genes revealed similar up- or down-expression trends and thus the results based on *ACTIN2* were selected. The log_2_ change in transcript abundance was calculated to allow for direct comparison with the RNA-seq data.

## Results

### Disease symptom development and tissue sampling of infected plants

**Fig. 1:**
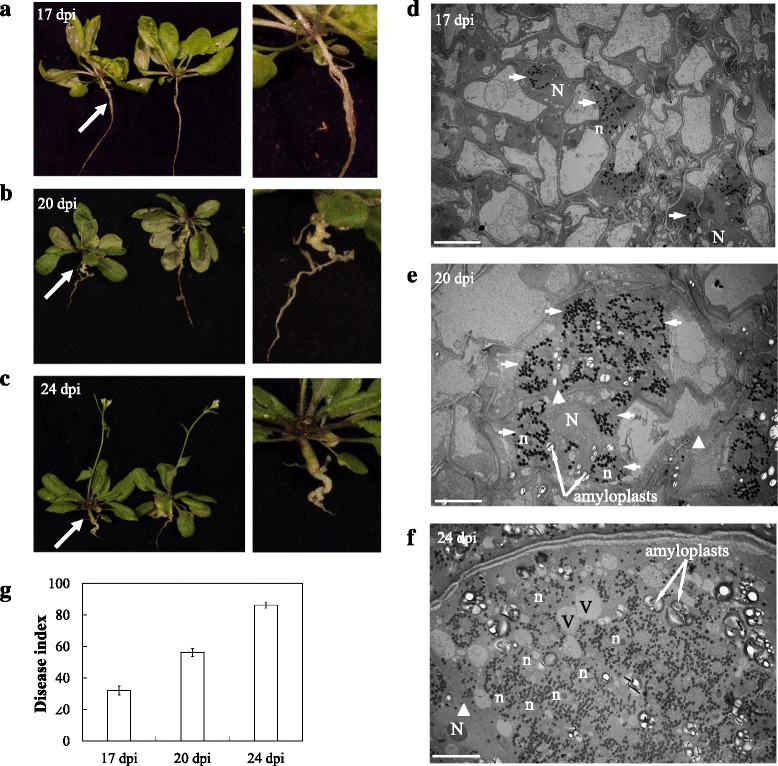
**a-c** Phenotype of roots in Arabidopsis plants infected by *P. brassicae*. **a** Initial symptoms of pathogen infection in roots by swelling at 17 dpi. **b** Formation of visible galls in roots at 20 dpi. **c** Deformed roots with big galls expanded through the roots at 24 dpi. **d**-**f** TEM micrographs of ultrastructural features of *P. brassicae* development and host cell showing magnification of the area indicated by the white arrows. **d** Uninucleate secondary plasmodium (white arrow) with abundant lipid droplets (black dots) in cortical cells at 17 dpi. **e** Multiple secondary plasmodia with abundant lipid droplets in cortical cells at 20 dpi. Thin or broken cell walls are shown by white arrowheads. **f** Multinucleate secondary plasmodia in the host cell at 24 dpi. Thin cell wall is shown by a white arrowhead. Amyloplasts/starch granules and small vacuoles are spotted. N: host nucleus; n: pathogen nucleus; v: vacuole. Scale bar: 10 μm. **g** Disease index of Arabidopsis plants at 17, 20 and 24 dpi. The data are the mean of three independent experiment. Error bars show the standard error of mean (SEM)

To determine the appropriate time points at which to perform RNA-seq analysis, we inoculated Arabidopsis seedlings by adding resting spore suspensions of *P. brassicae* to the soil and monitored disease establishment. During an infection time course, swelling of the primary and lateral roots was the first visible disease symptom observed on most roots between 16 and 18 dpi (Fig. 1a). At 20 dpi, galls were clearly visible on the primary and lateral roots (Fig. 1b), and by 24 dpi, most roots were deformed with galls spreading along the roots (Fig. 1c). At 24 dpi, the lateral roots were very fragile and often pulled away from the primary root during washing to remove the soil. Leaves of diseased plants started to show visible symptoms of stunting at 20 dpi, followed by wilting and turning yellow and/or purple (Additional file 2: Figure S1).

We used TEM to evaluate pathogen progress at 17, 20 and 24 dpi in infected root tissue. At 17 dpi, uninucleate secondary plasmodia were often observed in cortical cells (Fig. 1d). At 20 dpi, multiple secondary plasmodia were found in infected cortical cells. At this infection stage, the cells were enlarged with irregular shapes and the cell wall was thin compared to 17 dpi. Moreover, amyloplasts accumulated in infected cells at 20 dpi (Fig. 1e). At 24 dpi, pathogenesis advanced further in the host cells, resulting in the formation of multinucleate secondary plasmodia, that filled the giant infected cortical cell (Fig. 1f). In all time points, lipid droplets were abundant in the plasmodia in infected cells. At time points beyond 24 dpi, TEM showed that infected host tissues were degenerating with the disintegration of cell organelles, including rough endoplasmic reticulum. These deteriorating cells would be unable to produce an active defence response and as such our RNA-seq analysis did not go beyond the 24 dpi reported herein.

Disease severity of inoculated plants was further quantified by determining the disease index (DI) at 17, 20 and 24 dpi (Fig. 1g), with each time point primarily associated with initial visible symptoms of pathogen infection in roots (17 dpi), gall formation in roots (20 dpi), and large root galls with severe phenotypic symptoms on aerial parts of plants (24 dpi). The DI showed the progression of disease severity through these time points from 17 dpi (DI 32) to 20 dpi (DI 56) and 24 dpi (DI 86) (Fig. 1g). At these time points, above- and below-ground tissues of infected plants and mock control plants were collected for RNA-seq analysis.

### Overview, mapping and validation of RNA-seq data

**Fig. 2:**
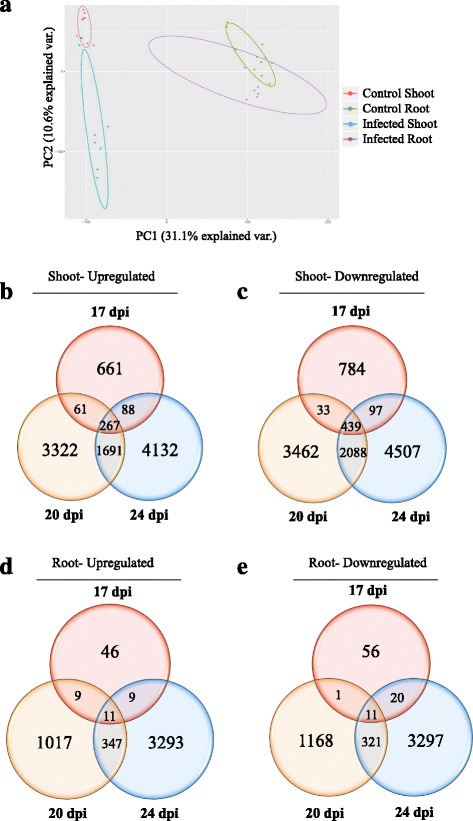
**a** PCA plot displaying biological variation between infected and control samples in shoot and root. **b-e** Venn diagrams showing the total number of significantly DEGs (*p*-value ≥0.05) at 17, 20 and 24 dpi in infected shoot and root compared to mock-infected control samples. The overlapping regions correspond to the number of DEGs present at more than one time point. **b** Up-regulated DEGs in shoot **c** Down-regulated DEGs in shoot. **d** Up-regulated DEGs in root **e** Down-regulated DEGs in root

RNA-seq analysis was performed on shoot and root tissues of *A. thaliana* for three biological replicates at each time point (17, 20 and 24 dpi), with resting spores of *P. brassicae* or mock inoculation as control. Approximately, 478 million (M) reads for infected samples (232 M for shoot and 246 M for root) and 458 M reads for control samples (214 M reads for shoot and 243 for root) were obtained (Additional file 2: Figure S2A). Almost 94% of the reads were mapped to the reference *A. thaliana* genome (Additional file 2: Figure S2A). Figure 2a shows principle component analysis (PCA) of the transcriptome FPKM values for each sample in each group (infected shoot, infected root, control shoot, control root). As expected, a similar expression pattern of some genes in both control and infected samples was observed. PCA also showed more separation between control and infected shoot samples compared to control and infected root. PCA analysis identified one of the infected root replicates (rep 2, at 17 dpi) grouped with the control shoot group. This misplaced point suggests that this sample was either contaminated or there was a technical issue during RNA-seq analysis. A revised PCA plot without data from the second replication of infected root at 17 dpi was prepared and shown in Additional file 2: Figure S2B.

To identify Arabidopsis genes that were significantly up- or down-regulated in infected tissues compared to corresponding controls, DeSeq2 was used to generate log_2_ fold change values for each gene. Volcano plots were used to provide a global view of distribution of DEGs in each tissue and time point (Additional file 2: Figure S3). Inoculation of *A. thaliana* with *P. brassicae* resulted in more changes in transcript levels in the shoot (Fig. 2a-b) relative to the root (Fig. 2c-d). Moreover, with disease progression, the number of differentially expressed genes (DEGs) increased in both infected tissues (Fig. 2). Thus, the plant transcriptomic response to clubroot disease was most strongly activated at 24 dpi. However, while the number of DEGs was lower at 17 dpi, compared to 20 and 24 dpi, these genes may represent critical early genes in the plant defense response before disease progression severely affects the plant. In addition, there were more DEGs with high fold changes (both up- or down-regulated) in shoot compared to root (Additional file 2: Figure S3).

**Fig. 3:**
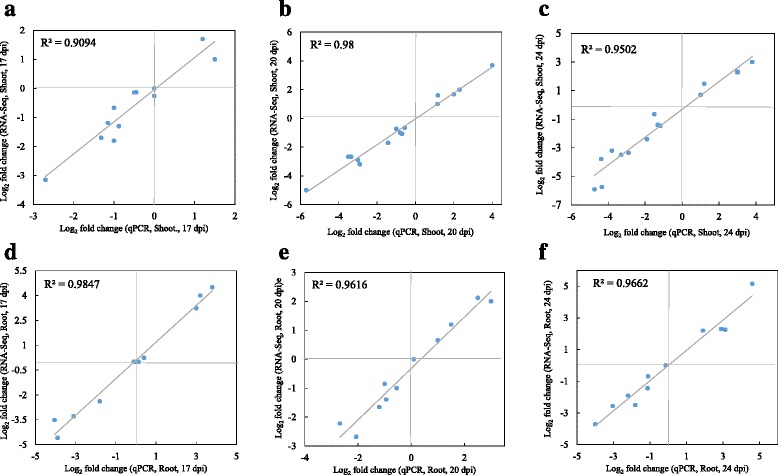
Comparison of gene expression values obtained by RNA-seq and Real-time PCR (qPCR)^.^ The RNA-seq log_2_ values of the expression ratio (infected versus uninfected) (y-axis) was compared to the same values from the qPCR (x-axis). The R2 values show the correlation ratio between RNA-seq and qPCR in each tissue and time point. *p*-value ≤0.05. **a** Shoot, 17 dpi **b** Shoot, 20 dpi **b** Shoot, 24 dpi **d** Root, 17 dpi **e** Root, 20 dpi **f** Root, 24 dpi

To validate the RNA-seq data, transcript levels of 15 genes in shoot and 11 genes in root were determined with qRT-PCR. Candidate genes for qRT-PCR analysis were chosen as representative of the up or down-regulated categories based on their up and down fold-change values. To directly compare RNA-seq data and qRT-PCR data, the relative fold change in expression level of genes obtained by qRT-PCR was converted to log_2_ (fold change). The resulting changes in expression of selected genes showed up- or down-regulation trends similar to those from the RNA-seq data (Fig. 3).

### Highly differentially regulated genes (DEGs) in infected shoot and root

GO enrichment analysis was performed on the DEGs in each tissue to map them to biological processes. DEGs, in root and shoot at 24 dpi, with at least ±1 log_2_ (fold change) and *P* ≤ 0.05 were selected for GO analysis (Additional file 2: Figure S4). In shoot, the highest percentage of up-regulated genes were mapped to the regulation of biological processes and to stress responses while the highest percentage of down-regulated genes were mapped to metabolic processes, cellular biogenesis and signal transduction. In root, up-regulated genes mapped to the response to stimuli and stress and lipid and cyclic compound metabolic processes, while highly down-regulated genes mapped to macromolecule modification/metabolic processes and cell wall organization (Additional file 1: Figure S4).

**Fig. 4:**
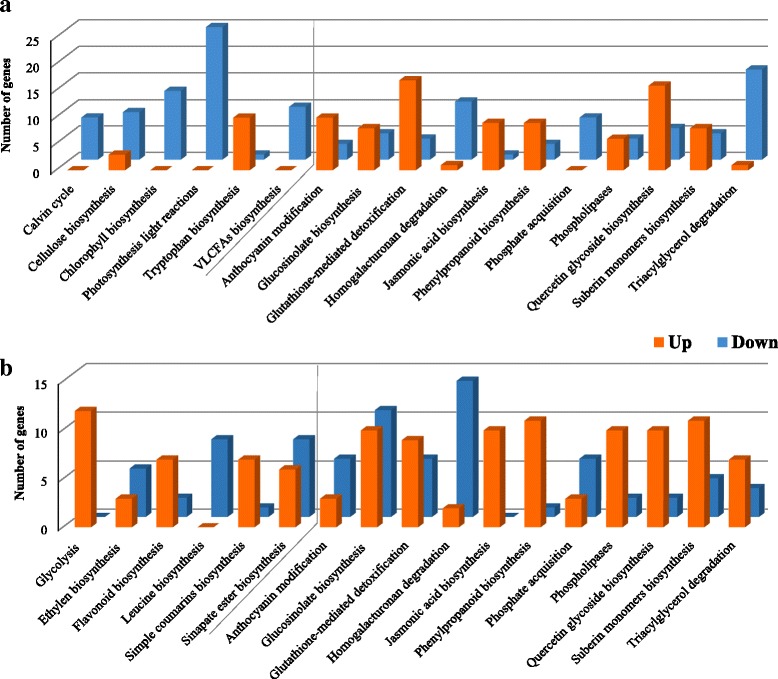
Identification of highly DEGs and their related pathways in infected shoot (**a**) and root (**b**) at 24 dpi. The right side of the gray line shows the pathways that are common between shoot and root. The y-axis shows the number of genes that were highly regulated. Columns in red are up-regulated and in blue are down-regulated genes. VLCFAs; very long chain fatty acids

In order to characterize the specific pathways that are differentially regulated, highly DEGs were mapped to pathways using ver. 10 of the TAIR pathway annotations, resulting in identification of pathways with highly up- or down-regulated genes at 24 dpi (Fig. 4). Highly up- and down-regulated pathways for 17 and 20 dpi can be found in Additional file 2: Figures S5 and S6.

At 24 dpi, 17 highly regulated pathways, in which at least eight genes were highly up- or down-regulated, were identified. As shown in Fig. 4 (right side of the gray line), 11 of 17 (64%) of the most highly up- or down-regulated pathways were common to both tissues. Three of the 11 pathways (anthocyanin modification, glucosinolate biosynthesis, and triacylglycerol degradation) were oppositely up- or down-regulated in the two tissues, with the remaining eight pathways regulated in the same direction in both root and shoot tissues (Fig. 4, right side of the gray line). As expected, in the shoot, genes involved in photosynthesis light reactions were the most down-regulated at 20 and 24 dpi. Triacylglycerol degradation genes, were the next most down-regulated DEGs in the shoot at both 20 and 24 dpi (Fig. 4a. Additional file 2: Figure S6). At 20 dpi, five highly regulated pathways are common between shoot and root (Additional file 2: Figure S6). Of these, three (kaempferol glycoside biosynthesis, quercetin glycoside biosynthesis, and triacylglycerol degradation) were oppositely up- or down-regulated in the two tissues. At 17 dpi, sinapate ester biosynthesis was the only common highly (up) regulated pathway in both shoot and root, with no common down-regulated pathways identified (Additional file 2: Figure S5). With disease progression, the number of genes and pathways differentially regulated in both tissues increased, with more common pathways emerging, in shoot and root, with disease establishment in the root between 20 and 24 dpi.

The critical roles of secondary metabolites in plants is reflected by the growing number of genes identified in the Arabidopsis genome that contribute to secondary metabolism [33]. In both infected shoot and root, the expression of genes downstream of the shikimate pathway, including the biosynthesis of flavonoids (shoot 17 & 20 dpi, root 24 dpi), phenylpropanoids (shoot 24 dpi, root 20 & 24 dpi), glucosinolates (shoot 24 dpi, root 20 & 24 dpi), quercetin glycosides (shoot 20 & 24 dpi, root 20 & 24 dpi,), kaempferol glycosides (shoot & root, 20 dpi) and simple coumarins (root 24 dpi), were altered in response to disease progression (Fig. 4. Additional file 2: Figure S5 and S6). Many DEGs for cell wall-associated compounds were also identified: homogalacturonan degradation (down-regulated in root 24 dpi, shoot 20 & 24 dpi), suberin monomer biosynthesis (up-regulated in root 20 & 24 dpi, shoot 20 & 24 dpi) and cellulose biosynthesis genes (down-regulated in shoot 20 & 24 dpi). Genes associated with the degradation of triacylglycerols were mainly up-regulated in root but down-regulated in shoot at 20 and 24 dpi (Fig. 4. Additional file 2: Figure S6). In parallel, the biosynthesis of very long chain fatty acids (VLCFAs) was down-regulated in shoot at all three time points. Noticeably, in both shoot and root at 24 dpi, JA biosynthesis genes were highly up-regulated (Fig. 4). Likewise, in both shoot and root, glutathione-mediated detoxification pathway genes were up-regulated at 20 and 24 dpi (Fig. 4. Additional file 2: Figure S6). In 17 and 20 dpi root, several genes in the glutamate synthase pathway (GS-GOGAT cycle) were up-regulated, e.g., ammonia assimilation (ammonia to glutamine), glutamine biosynthesis pathway and glutamate-glutamine shuttle (Additional file 2: Figures S5 and S6).

### Metabolism overview

**Fig. 5:**
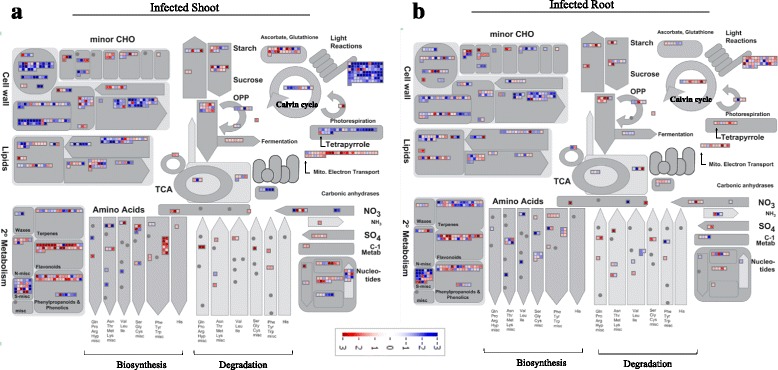
Overview of the transcriptional changes of genes involved in metabolism in root and shoot of *P. brassicae*-infected *Arabidopsis* plants at 24 dpi. MapMan analysis of DEGs in shoot (**a**) and root (**b**) of Arabidopsis plants in response to infection by *P. brassicae*, showing assignment to different metabolic compartments. Genes in red are up-regulated and in blue are down-regulated. The regulation of genes is based on log_2_ fold change. MapMan of the model plant *A. thaliana* was used

Analysis of the RNA-seq data with MapMan software enabled an overall metabolic view of the transcriptome data [34] (Fig. 5).

#### Amino acids

Regulation of amino acid metabolism, in response to clubroot infection and disease progression, has been previously reported [35, 36]. Here, aside from glutamine biosynthesis genes in root (Additional file 3), transcripts of amino acid biosynthesis genes were down-regulated in both root and shoot, whereas transcripts involved in amino acid degradation were induced (Fig. 5). Tryptophan was the exception, as its biosynthesis was highly up-regulated, especially in shoot tissue (Additional file 3).

#### Tetrapyrroles

Tetrapyrroles are abundant compounds in plants and include chlorophyll, heme, phytochromobilin, siroheme, and their derivatives. They serve important roles in photosynthesis, plastid to nucleus signalling, chloroplast development, and resistance to stress conditions [37]. Here, expression of tetrapyrrole synthesis genes was generally down-regulated in infected shoot. For example, by day 24, expression of almost all of tetrapyrrole synthesis genes were reduced in shoot (Additional file 3). In contrast, expression of tetrapyrrole synthesis genes remained unchanged or were slightly increased in root at 24 dpi.

#### Photosynthesis and respiration

Expression of nearly all genes encoding light reactions and carbon fixation (photosynthesis) were reduced in infected shoot (Fig. 5). Likewise, transcripts of most Calvin cycle genes and genes encoding the rubisco subunits were reduced in infected shoot (Fig. 5). Many tricarboxylic acid (TCA) cycle genes were down-regulated in shoot at 20 and 24 dpi (Additional file 3). In infected root, transcripts of Calvin cycle and TCA cycle genes were up-regulated (Additional file 3). Transcripts of genes in the mitochondrial electron transport pathway were enhanced in both shoot and root, particularly in shoot at 20 and 24 dpi (Additional file 3).

#### Starch and sucrose pathways

Previous reports suggest that both the starch and sucrose pathways have a role in *P. brassicae*-plant interactions in Arabidopsis [38, 39]. In our study, in infected shoot, DEGs involved in starch and sucrose biosynthesis had a mixed regulation (both up and down) at all three time points, especially at 24 dpi (Additional file 3). In infected root, expression of these genes was predominantly up-regulated at 20 and 24 dpi. In addition to starch and sucrose biosynthesis genes, several DEGs in the glycolysis pathway were up-regulated in root at 24 dpi (Additional file 3). In both root and shoot, the highest expression among the starch/sucrose transmembrane transporter genes was a glucose-6-phosphate/ phosphate translocator (*GPT2*; At1g61800) (Additional file 3). Other than this gene, 10 out of 12 differentially expressed starch/sucrose transporters in this group were down-regulated in infected shoot at 24 dpi. In root, all of nine differentially expressed transporters were up-regulated at 24 dpi. It is likely that following an increase in expression of starch degradation and glycolysis genes in infected root, up-regulation of a set of transmembrane transporter genes is also required to mobilize sucrose. Among fermentation genes, three pyruvate decarboxylase genes were relatively up-regulated in infected shoot at 24 dpi (Additional file 3).

#### Lipids

Genes related to fatty acid desaturation, synthesis, and elongation (including beta ketoacyl CoA synthase, fatty acid elongase beta hydroxyacyl, acyl carrier protein dehydratase and ketoacyl CoA synthase genes) were down-regulated in shoot, primarily at 24 dpi (Fig. 5; Additional file 3). Lipid genes were similarly down-regulated in root but fewer fatty acid biosynthesis and desaturation genes were affected at 24 dpi (Fig. 5). However, expression of several phospholipid synthesis genes was up-regulated in root at 17 and 20 dpi (Additional file 3). Moreover, several fatty acid (FA) synthesis and elongation genes were up-regulated in root at 20 dpi (Additional file 3). Fatty acid synthase genes were not identified in *P. brassicae*, indicating the dependence of the pathogen on the host for these compounds [5]. Higher expression of lipid synthesis genes in root at 17 and 20 dpi is probably linked to the accumulation of lipid droplets in plasmodia in infected root cells as shown at all time points (Fig. 1d-f).

#### Secondary metabolites

Expression of genes involved in several secondary metabolism pathways was widely affected by *P. brassicae* infection (Fig. 5). MapMan analysis revealed that the biosynthetic pathways (flavonoid and phenylpropanoid) downstream of the shikimic acid were highly up-regulated in both shoot and root tissues at 24 dpi. Notably, the transcript encoding a chalcone synthase (At5g13930, TRANSPARENT TESTA 4) showed the highest up-regulation among all DEGs at 24 dpi. Several genes involved in glucosinolate biosynthesis were down-regulated in the root, whereas regulation of these genes was more complicated in shoot (Additional file 3). In contrast to the up-regulation of genes in the downstream pathways of shikimic acid metabolism, terpene biosynthesis, via the intermediacy of the C_5_-isoprenoid precursors isopentenyl diphosphate (IPP) and dimethylallyl diphosphate (DMAPP), was significantly down-regulated in both infected shoot and root (with higher numbers of DEGs in the root). Two principle pathways that produce plant secondary products, i.e. shikimic acid metabolism and terpene specialized biosynthesis, appear to have been relatively well defined in higher plants. The discrepancy of the expression regulation of shikimic acid pathway and terpene metabolism in *P. brassicae*-infected plants suggests that the host metabolite flow into shikimic acid-derivatives may play important roles in clubroot disease development.

### Regulation of cell wall-related genes in response to *P. brassicae* infection

**Fig. 6:**
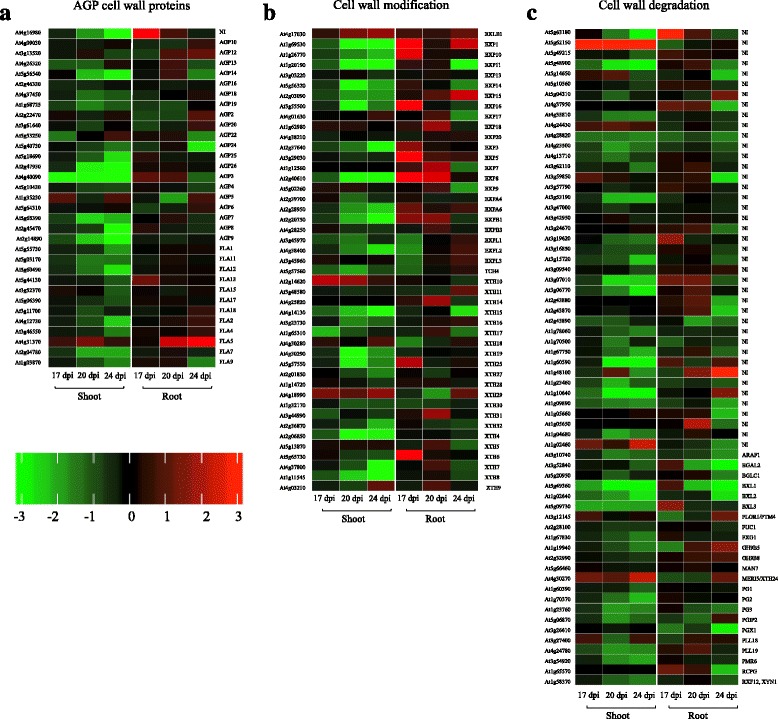
Plant cell wall proteins in responses to *P. brassicae* inoculation. Heat maps of DEGs encoding (**a**) cell wall proteins. AGPs (**b**) cell wall modification proteins (**c**) cell wall degradation proteins at 17, 20 and 24 dpi in shoot and root. Genes in red are upregulated and in green are downregulated. The regulation of genes is based on log2 fold change. The heat maps show the DEGs in infected tissues compared to the mock-infected control samples. *AGP* arabinogalactan-protein, *LRR* leucine-rich repeat, *NI* Not Identified (no specific gene name was listed for the locus in TAIR)

Changes in the transcript levels of many cell wall genes following inoculation confirms the major physical changes in plant structures occurring during disease progression (Fig. 5). To compare the variation in gene expression in each tissue at the three time points, heat maps of the major cell wall DEGs were prepared (Fig. 6). There was a down-regulation of many cell wall component genes especially arabinogalactan-proteins (AGPs; involved in cell division, expansion and leaf development [40]) in shoot at all post-inoculation time points (Fig. 6a). Genes encoding cell wall modification proteins were down-regulated in shoot tissues, but up-regulated in root tissues at 17 and 20 dpi (Fig. 6b). Many of the up-regulated cell wall modification genes in root are members of the alpha-expansin (EXP) gene family that were strongly induced at 17 and 20 dpi (Fig. 6b). Intriguingly, many transcripts encoding hydrolytic enzymes of cell wall degradation such as pectinases, glucanses and cellulase, were down-regulated in both shoot and root tissues of infected plants, with the most noticeable down-regulation at 20 and 24 dpi in shoot and at 24 dpi in root (Fig. 6c), suggesting that *P. brassicae* infection induces cell wall modifications rather than hydrolytic degradations of structural cell wall components of infected cells during clubroot establishment.

### Differentially expressed hormone biosynthesis and defence response genes

**Fig. 7:**
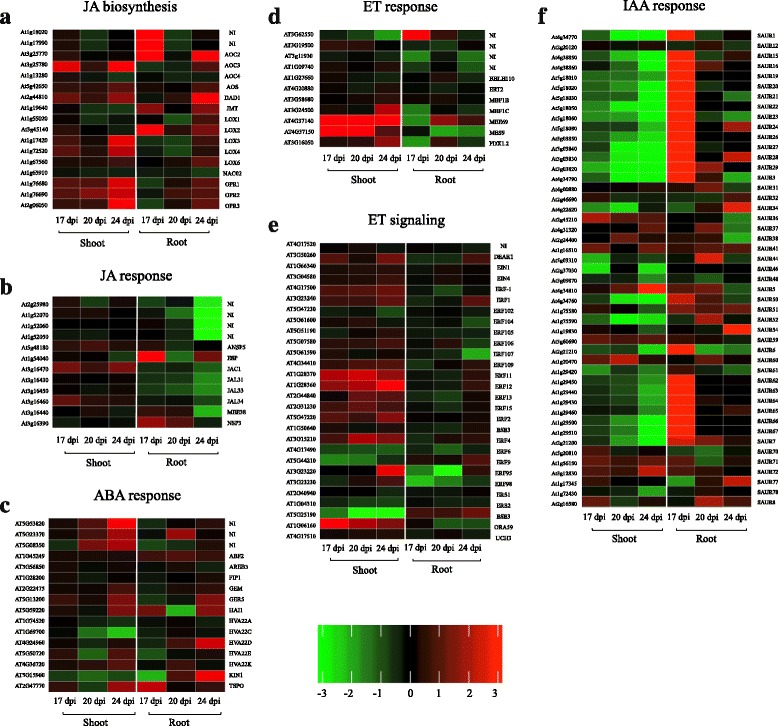
Plant hormones in responses to *P. brassicae* inoculation*.* Heat maps of DEGs encoding **a** JA biosynthesis, **b** JA response, **c** ET response, **d** ET signaling, **e** IAA response, **f** ABA response. Genes in red are up-regulated and in green are down-regulated. The regulation of genes is based on log_2_ fold change. The heat maps show the DEGs in infected tissues compared to the mock-infected control samples. ABA; abscisic acid. ET; ethylene. IAA; indole acetic acid (auxin). JA; jasmonic acid. NI: Not Identified (no specific gene name was listed for the locus in TAIR)

Changes in transcript levels for genes involved in hormone metabolism, response, and signaling were further evaluated in infected shoot and root tissues. Altogether, the results indicated that JA has an important role in the plant defense response to *P. brassicae* infection and clubroot establishment (Fig. 7a). Transcript levels from JA biosynthesis genes (e.g., 12-oxophytodienoic acid reductase, lipoxygenase, and allene oxidase cyclase) were generally up-regulated in shoot (Fig. 7a). A similar pattern of transcript accumulation was observed in root, with expression of many JA biosynthesis genes increased at 24 dpi, but not to the same level as observed in shoot (Fig. 7a). It is notable that transcript levels of JA response genes were down-regulated in root at 24 dpi (Fig. 7b). Abscisic acid (ABA) response genes were mostly up-regulated in both root and shoot at 24 dpi (Fig. 7c). In shoot, transcript levels of a number of ET response genes and several ET signaling genes were up-regulated starting at 17 dpi, with limited change in expression level in the root (Fig. 7d and e). Transcript levels of many small auxin upregulated RNA (*SAUR*) genes involved in IAA response pathway were highly down-regulated in shoot at 20 and 24 dpi but highly up-regulated in root 17 dpi, after which transcript levels decreased (Fig. 7f).

### Differentially expressed plant biotic stress response genes

Several genes related to defense signaling and responses were differentially expressed in infected tissues. Not surprisingly, the number of significantly DEGs and the fold changes were higher in root than in shoot (Additional file 3). Biotic stress related genes were mostly regulated in 17 dpi tissues compared to the later 20 and 24 dpi time points. In plants, the majority of known disease resistance genes (R genes) encode Nucleotide-Binding Site-Leucine-Rich Repeat (NBS-LRR) proteins [41]. In root, seventeen genes encoding NBS-LRR proteins were induced with log_2_ (fold change) ≥1, at 17 dpi (Additional file 3). The other class of R genes, Receptor-Like Proteins (RLPs) are cell-surface receptors with roles in plant disease resistance [42]. In addition, plant genomes encode many Receptor-Like Kinases (RLKs) that function in (pathogen) elicitor-activated defense response and as R genes in response to pathogen attack [43]. Several RLP and RLK protein genes were up-regulated in root at 17 dpi (Additional file 3). Transcript levels for one RLP and five RLKs were also increased in shoot at 17 dpi but with smaller fold changes. Plant defensins are small cysteine-reach peptides that are involved in defense against pathogen infection [44]. Transcript levels for eight plant defensin genes, including the well reported *PDF1.2* (At5g44420) and *PDF1.3* (At2g26010) were up-regulated in root at 17 dpi. Likewise, higher abundance of a few SA-responsive pathogenesis-related gene transcripts was found in root at 17 dpi (Additional file 3). Intriguingly, the up-regulation of biotic stress-related genes occurs only at the early 17 dpi time point and not at the later 20 and 24 dpi time points. At 17 dpi, *P. brassicae* infected plants began to show initial disease symptoms in roots, with slightly swollen main roots and no abnormal appearance on above ground tissues, whereas infected plants at 20 and 24 dpi showed abundant gall formation on roots and severe phenotypic symptoms on above ground tissues. The expression pattern of the defense responsive genes indicates a switch point in host tissues from an initial signalling/defensive response at 17 dpi to the establishment of a susceptible interaction at 20–24 dpi with clubroot development.

### Regulation of TF genes in response to infection

TFs are the key to the initiation and regulation of gene transcription. Members of several TF families (e.g., MYB and WRKY) play important roles in the regulation of defense genes in response to pathogen attacks [45]. In this study, expression of 143 (66 up- and 77 down-regulated) and 61 (41 up and 20 down) TF genes was altered at least ±2 log_2_ (fold change) in shoot and root, respectively, over the 17, 20 and 24 dpi time points (Table 1). Among these, thirteen TF genes were co-regulated (seven up and six down) in infected root and shoot, and two genes showed opposite expression, up in shoot and down in root (see the genes’ ID in Table 1). The identified genes were grouped into 18 different families in shoot, with representatives of only 15 of these families in root. Without considering the common DEGs between three time pointes, at 24 dpi, expression of 143 TF genes (58 up- and 85 downregulated) was affected in infected shoot, whereas at the 17 and 20 dpi time points together, the expression of only 36 and 48 TF genes were up- and down-regulated, respectively. In root, at 24 dpi, the number of TF genes showing at least a log_2_ ± 2 fold change was 54 (34 up- and 20 down-regulated), with only three TF genes down-regulated and 19 TF genes up-regulated at the 17 and 20 dpi time points combined.

**Table 1:** DEGs belonging to TF families with log_2_ fold change ≥ ± 2 at three tested time points

TF family	Total number	Common expression	Opposite expression	Change	Shoot	Root
AP2/EREBP	17	0	0	Up	5	4
				Down	7	1
Aux/IAA	2	0	0	Up	0	0
				Down	2	0
B3 (DBP)	6	1 (Up) At1g49475	0	Up	1	2
				Down	3	0
bHLH	36	1 (Up) At4g29930	0	Up	9	7
				Down	17	3
bZIP	8	0	0	Up	4	1
				Down	2	1
C2C2-CO-like	8	1 (Up) At3g21890	1 (Up in shoot, Down in root) At3g21150	Up	4	1
				Down	2	1
C2H2	9	1 (Up) At3g53600	0	Up	2	1
				Down	6	0
C3H	5	2 (Down) At5g44260 At2g25900	0	Up	1	0
				Down	2	2
CCAAT	8	2 (Up) At3g05690 At5g06510	0	Up	2	5
				Down	1	0
G2-like	4	1 (Down) At4g37180	0	Up	0	0
				Down	2	2
HB	11	0	0	Up	0	4
				Down	6	1
HSF	4	0	0	Up	3	1
				Down	0	0
LOB	8	1 (Down) At4g37540	0	Up	2	1
				Down	4	1
MYB	39	1 (Up) At5g56840	0	Up	20	9
				Down	7	3
Orphans	4	0	0	Up	2	0
				Down	2	0
Putative	8	0	0	Up	2	1
				Down	3	2
Unclassified	21	2 (Down) At1g32540 At4g01335	1(Up in shoot, Down in root) At2g05160	Up	3	4
				Down	11	3
WRKY	6	0	0	Up	6	0
				Down	0	0

Collectively, in both root and shoot, the highest number of affected TF genes (log_2_ fold change ≥ ± 2) was found in the MYB (27 shoot, 12 root; with one common to both tissues) and bHLH (26 shoot, 10 root; with one common to both tissues) families followed by the AP2/EREB (12 shoot, 5 root) family. Twenty-one TFs (14 shoot, 7 root; with three common to both tissues) were unclassified within these 18 families.

TFs with roles in plant hormone-responsive transcription were also identified, with the highest number of DEGs involved in the ethylene response pathway. The hormone-responsive TFs with at least log_2_ ± 2 fold change in expression at the three time points are listed in Additional file 3.

## Discussion

As a biotrophic pathogen, *P. brassicae* has likely evolved several strategies to avoid host recognition and subsequent cell death to establish an intracellular parasitism within living host cells, enabling it to extract/absorb nutrients from its host and to direct cell proliferation of host root tissues to produce a nutrient-sink gall habitat. There is currently limited resistance germplasm in the Brassicaceae, so exploring and understanding the genomics of disease progression in this host plant will contribute to the development of a clubroot resistance breeding strategy for canola.

Our research provides a broad-spectrum study of the Arabidopsis transcriptomic response to *P. brassicae* inoculation, in above- and below-ground tissues of the host plant. As expected, we observed more DEGs at later stages of the disease establishment, with the largest transcriptomic changes occurring in the 24 dpi samples of both shoot and root. Although the root is the infected organ, the number of DEGs and the fold change in the DEGs, across all three time points sampled was higher in the shoot (Fig. 2b-e), suggesting that metabolic and cellular processes of the shoot are in fact more affected by clubroot disease progression than those of the infected root. A higher number of DEGs in the shoot is probably a result, in some part, of a general stress response to the disease, as shown by the down-regulation of photosynthesis and light reaction genes (Figs. 4a and 5a). Whereas, gene expression in diseased roots would predominantly be a specific response to the pathogen attack. Few studies have analyzed the shoot response to root pathogen attack. However, a similar down-regulation of genes involved in photosynthesis, together with an up-regulation of pathogen protection and oxidative stress genes, has been reported in shoots in response to inoculation of roots with rhizobacterium [46]. Although spatially separated, the above- and below-ground tissues of plants do interact with each other and our results suggest that future studies are needed to address this interaction in response to root pathogens.

The identification and functional classification of highly DEGs showed that the expression of many of these genes was co-regulated or oppositely regulated in shoot and root. As expected, in the shoot, DEGs involved in photosynthesis were down-regulated after root inoculation with *P. brassicae* and subsequent clubroot progression (Fig. 4a). Such a down-regulation has previously been reported for genes involved in photosynthesis, biosynthesis of chlorophyll and the small subunit of rubisco in response to pathogen attacks [47, 48]. Furthermore, several genes involved in the degradation of starch and triacylglycerols (lipid storage molecules) and the Calvin cycle were also down-regulated in the shoot (Fig. 4a). Following decreased water and nutrient uptake by the host plant, the general down-regulation of these genes in the shoot of infected plants is predictable. A decrease in starch and soluble carbohydrates in the leaves of Arabidopsis, five weeks after infection with *P. brassicae*, has previously been reported [49]. These findings were compatible with a reduced rate of photosynthesis in Arabidopsis leaves [49]. Contrary to these decreases, tryptophan (shikimate pathway metabolite) biosynthesis genes were highly induced in infected shoot, especially at 24 dpi (Additional file 3). Increased levels of tryptophan-derived metabolites (e.g., camalexin and indol glucosinolates) have been reported in a number of plant species in response to various pathogen infections/disease progression [50, 51]. Increased transcript levels for *CHALCONE SYNTHASE* (*CHS*) were also found in the shoot at 20 and 24 dpi (Additional file 3). Under stress conditions, chalcone compounds produced through the shikimate pathway, serve as antioxidants and antimicrobials (phytoalexins) [52].

Infected Arabidopsis plants exhibited impaired growth and induced senescence, particularly at 20 and 24 dpi (Additional file 2: Figure S1). Over this time period, the genes encoding cell wall structural proteins were mainly down-regulated in infected shoot (Fig. 6a). In addition, expression of many cell wall modification and degradation genes were turned off in the shoot over the 17, 20 and 24 dpi time points (Fig. 6b-c). ET signaling genes were induced in shoot following inoculation, peaking at 24 dpi (Fig. 7e). Depending on the pathogen and the host plant, ET signaling can play different roles during plant disease [53]. One of the main suggested functions for ET in response to non-necrotrophic pathogens is induction of programmed cell death [53].

In infected root, expression of starch and sucrose biosynthesis and glycolysis pathway genes was mainly up-regulated (Additional file 3). An increase in various sugars and starch has been previously reported in *P. brassicae* infected Arabidopsis roots [38, 49], whereas a suppression of invertase expression in transgenic plants resulted in enhanced tolerance to *P. brassicae* [39]. The current results indicate an increased availability of carbon sources at the infection sites as a way to resource the metabolic activities of the pathogen as well as the increased cell division of the host plant, resulting in galls at these sites. Furthermore, DEGs involved in lipid biosynthesis and elongation were up-regulated at 17 and 20 dpi in root. The higher abundance of these genes might be a response for the pathogen lipid demand. An up-regulation of lipid transfer genes was found in infected Arabidopsis roots when galls and resting spores were present, suggesting the formation of a lipid reserve [9]. This is consistent with our observation of abundant lipid droplets in the pathogen cytoplasm (Fig. 1d-f). Increased expression of phospholipases in infected root, mainly at 24 dpi (Fig. 4b), may be attributed to their roles in the synthesis of JA, oxylipins and other important plant defense signaling molecules [54]. However, since phospholipases hydrolyze phospholipids, an increased expression may indicate an increased level of catabolization of phospholipids by the host plant.

A group of cell wall modification proteins, including expansins, xyloglucan endotransglucosylase/hydrolases (XTHs) and endo-β-1, 4-glucanases (EGases) facilitate cell wall expansion and function in regulating the plasticity and rheology of the cell wall [55]. The up-regulation of cell wall modification genes in the root is likely linked to cell wall loosening and cell expansion in infected root (Fig. 6b). However, several DEGs encoding cell wall degradation enzymes were strongly down-regulated in infected root at 24 dpi (Fig. 6c). These results suggest that *P. brassicae*-induced regulation of cell wall modification proteins is involved in an alteration of cell wall structure during infection. Moreover, in root, an up-regulation of *SAUR* genes that are an early auxin-responsive gene family, (Fig. 7f) is consistent with the suggestion that IAA induces cell division and elongation during gall formation in *P. brassicae*-infected Arabidopsis [7]. An involvement of auxin in the host - *P. brassicae* interaction has been previously reviewed by Ludwig-Müller [56].

In both 24 dpi shoot and root, the expression of several genes in major branches of the shikimate pathway was up-regulated (Additional file 2). At 17 dpi, genes involved in the biosynthesis of sinapate esters through the phenylpropanoid branch of the shikimate pathway were up-regulated in both tissues (Additional file 2: Figure S5). Both a higher level of transcription of shikimate pathway genes, together with an increased production of the pathway’s metabolites in plants, has previously been reported in response to pathogenic attacks [57]. In *P. brassicae* infected roots, an accumulation of phenylpropanoid pathway compounds [16, 58] or a higher expression of related genes [9, 14–16, 59] has been reported. Our results indicate that shikimate pathway metabolites have important roles in both root and shoot responses in infected host plants; however, further work is needed to assess if the upregulation of specific secondary metabolite genes in the shoot is an active response to the pathogen infection or an indirect negative effect of the disease on the shoot.

Transcriptome levels for genes in the JA biosynthesis pathway were elevated at all three time points in shoot and at 24 dpi in root (Fig. 7a). However, several genes involved in the JA response pathway were strongly down-regulated in root at 24 dpi, perhaps by the pathogen (Fig. 7b), indicating that the transcript regulation of JA response genes might be manipulated by the pathogen. It has previously been suggested that *P. brassicae* is able to manipulate a number of hormone pathways and can alter hormone homeostasis in the host plant to its benefit [5, 17] On the other hand, JA response genes may be down-regulated once the pathogen is established and having successfully disarmed the plant immune response by 24 dpi. An enhanced JA level was reported, in Chinese cabbage roots, three to five weeks after infection with *P. brassicae* [60]. High levels of jasmonate-related compounds was also reported in Arabidopsis ecotype Col-0 roots, 21 days after inoculation with *P. brassicae* [61]. In addition, during secondary infection by *P. brassicae*, the JA pathway was induced in susceptible Arabidopsis Col-0 but not in the partially resistant ecotype, Bur-0 [62]. Our results suggest that the JA pathway is involved in clubroot disease during *P. brassicae* secondary infection.

A number of DEGs in the ABA response pathway were up-regulated in both shoot and root at 24 dpi. The level of ABA has been reported to increase in *P. brassicae* infected *B. rapa* during gall formation [63]. Moreover, up-regulation of ABA response and signaling genes during late time points after *P. brassicae* infection has been reported [9, 10]. Up-regulation of ABA response genes might be a response to abiotic stress conditions in infected tissues that are subsequently under water and nutrient stress. However, it has also been proposed that ABA plays a role in the induction of plant defense response genes and JA-induced defense genes [64].

Members of the TF families, MYB, bHLH and AP2/EREBP, were highly up- or down-regulated in both shoot and root (Table 1). In Arabidopsis, the functions of MYB TFs are very diverse, including anthocyanin and flavonol biosynthesis, terminal differentiation and cell cycle regulation [65]. Similarly, members of the bHLH family play various roles in the regulation of ABA and gibberellin signalling pathways, stomata development and root development in Arabidopsis [65]. The comparison of expression profiles of TFs, in shoot and root tissues, highlighted the major differences in response of each tissue to the infection. As shown in Table 1, only a few TFs were commonly up- or down-regulated in both infected shoot and root.

## Conclusions

**Fig. 8:**
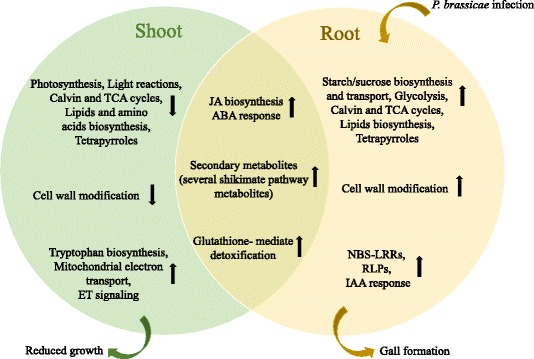
A simplified model to describe the major transcriptional regulation in shoot and root of *P. brassicae*-infected Arabidopsis. The overlapping region corresponds to common DEGs in shoot and root. The upward black arrows show up-regulated DEGs and the downward arrows show down-regulated DEGs. ABA; abscisic acid. ET; ethylene. IAA; indole acetic acid (auxin). JA; jasmonic acid. RLPs; Receptor-Like Proteins

Our data indicate that Arabidopsis has a complex transcriptomic response to *P. brassicae* infection, and in general, the plant response to pathogen infection of the root is different between shoot and root. Based on these results, we propose a simplified model of the major molecular changes in shoot and root in response to *P. brassicae* infection and clubroot disease progression (Fig. 8). In the root, there is a general induction of genes related to cell wall modification, starch/sucrose biosynthesis and transport to provide energy. In contrast, in the shoot, and possibly as a result of the impairment of nutrient and water uptake, we see a down-regulation of photosynthesis, chlorophyll synthesis, starch/sucrose biosynthesis and an inhibition of synthesis of proteins and enzymes involved in cell wall formation and modification. As a result, the leaves show symptoms of stress such as reduced growth and necrosis. However, the shoot and root also respond to the pathogen in a number of common ways, such as an induction of JA biosynthesis, ABA response and the production of shikimate pathway metabolites. Overall, our results describe an unprecedented expression dataset describing the shoot and root response of Arabidopsis to *P. brassicae* infection and clubroot disease.

## Additional files

Additional file 1: List of primers that were used for qRT-PCR analysis. (XLS 30 kb)

Additional file 2: **Figure S1.** Phenotype of Arabidopsis control and *P. brassicae*-infected plants at 20 and 24 dpi. Representative infected plants show disease symptom development as yellowish and/or purple and stunted leaves. Some lower leaves are undergoing cell death and are wilted. **Figure S2.** (A) Bar graph summarizing the number of mapped and unmapped RNA-seq reads in shoot and root in mock-infected control and *P. brassicae*-inoculated tissues in three independent replicates at 17, 20 and 24 dpi. The y-axis shows the number of reads per million. Red color presents the mapped and blue presents the unmapped data. (B) Revised PCA plot without second replication of infected root at 17 dpi. **Figure S3.** (A) Volcano plots for DEGs in shoot and root at 17, 20 and 24 dpi with variable Y-scale between plots (B) Volcano plots with a constant Y-scale (threshold at Y = 50) between plots. In volcano plots, black color represents a fold change with an absolute value less than or equal to 2. Dark blue represents a fold change with an absolute value less than or equal to 2. Purple color shows a fold change with an absolute value greater than 2. Green shows a fold change with a value less than −2, and. Red color represents a fold change with a value greater than 2. In all cases, *p*-value ≤0.05.**Figure S4.** GO analyses of DEGs in shoot and root at 24 dpi. The y-axis shows the percentage of genes mapped by the biological process term. A) GO terms for shoot. B) GO terms for root. **Figure S5.** Identification of highly DEGs in infected shoot (A) and root (B) at 17 dpi. The right side of the gray line shows the pathway that is common between shoot and root. The y-axis shows the number of highly regulated genes. Columns in red represent upregulated genes and in blue represent downregulated genes. VLCFAs; very long chain fatty acids. **Figure S6.** Identification of highly DEGs in infected shoot (A) and root (B) at 20 dpi. The right side of the gray line shows the pathways that are common between shoot and root. The y-axis indicates the number of highly DEGs. Columns in red and blue represent the upregulated and downregulated genes, respectively. VLCFAs; very long chain fatty acids. (PPTX 1073 kb)

Additional file 3: List of DEGs in several metabolism pathways and biological processes in Arabidopsis after *P. brassicae* infection. *P*-value was assigned as NA if there were not three counts for the gene (three replications) or the gene read contained an extreme count in one or more replicates. (XLSX 35 kb)

## Abbreviations

ABA: Abscisic acid
AGPs: Arabinogalactan-proteins
CK: Cytokinin
DEGs: Differentially expressed genes
DI: Disease index
dpi: Days post inoculation
ET: Ethylene
EXP: Expansin
FA: Fatty acid
FPKM: Fragments per kilobase of transcript per million mapped reads
GO: Gene ontology
GS-GOGAT: Glutamine synthetase-Glutamine oxoglutarate aminotransferase
IAA: Indole-3-acetic acid
JA: Jasmonic acid
M: Million
NBS-LRR: Nucleotide-binding site-leucine-rich repeat
PB: Phosphate buffer
PCA: Principle component analysis
qPCR: Quantitative PCR
RLKs: Receptor-like kinases
RLPs: Receptor-like proteins
SA: Salicylic acid
SAUR: Small auxin upregulated RNA
TAIR: The Arabidopsis information resource
TCA: Tricarboxylic acid
TEM: Transmission electron microscopy
TF: Transcription factor
VLCFAs: Very long chain fatty acids
