# Supplementary material for: Transcriptome analysis of response to Plasmodiophora brassicae infection in the Arabidopsis shoot and root
Source: BMC Genomics. 2018 Jan 5;19:23. doi: 10.1186/s12864-017-4426-7 (PMC5756429; doi:10.1186/s12864-017-4426-7)
Supplement: Supplementary file 2 — Figure S1. Phenotype of Arabidopsis control and P. brassicae-infected plants at 20 and 24 dpi. Representative infected plants show disease symptom development as yellowish and/or purple and stunted leaves. Some lower leaves are undergoing cell death and are wilted. Figure S2. (A) Bar graph summarizing the number of mapped and unmapped RNA-seq reads in shoot and root in mock-infected control and P. brassicae-inoculated tissues in three independent replicates at 17, 20 and 24 dpi. The y-axis shows the number of reads per million. Red color presents the mapped and blue presents the unmapped data. (B) Revised PCA plot without second replication of infected root at 17 dpi. Figure S3. (A) Volcano plots for DEGs in shoot and root at 17, 20 and 24 dpi with variable Y-scale between plots (B) Volcano plots with a constant Y-scale (threshold at Y = 50) between plots. In volcano plots, black color represents a fold change with an absolute value less than or equal to 2. Dark blue represents a fold change with an absolute value less than or equal to 2. Purple color shows a fold change with an absolute value greater than 2. Green shows a fold change with a value less than −2, and. Red color represents a fold change with a value greater than 2. In all cases, p-value ≤0.05.Figure S4. GO analyses of DEGs in shoot and root at 24 dpi. The y-axis shows the percentage of genes mapped by the biological process term. A) GO terms for shoot. B) GO terms for root. Figure S5. Identification of highly DEGs in infected shoot (A) and root (B) at 17 dpi. The right side of the gray line shows the pathway that is common between shoot and root. The y-axis shows the number of highly regulated genes. Columns in red represent upregulated genes and in blue represent downregulated genes. VLCFAs; very long chain fatty acids. Figure S6. Identification of highly DEGs in infected shoot (A) and root (B) at 20 dpi. The right side of the gray line shows the pathways that are common between shoot and root. The y- [file 12864_2017_4426_MOESM2_ESM.pptx]

## Slide 1
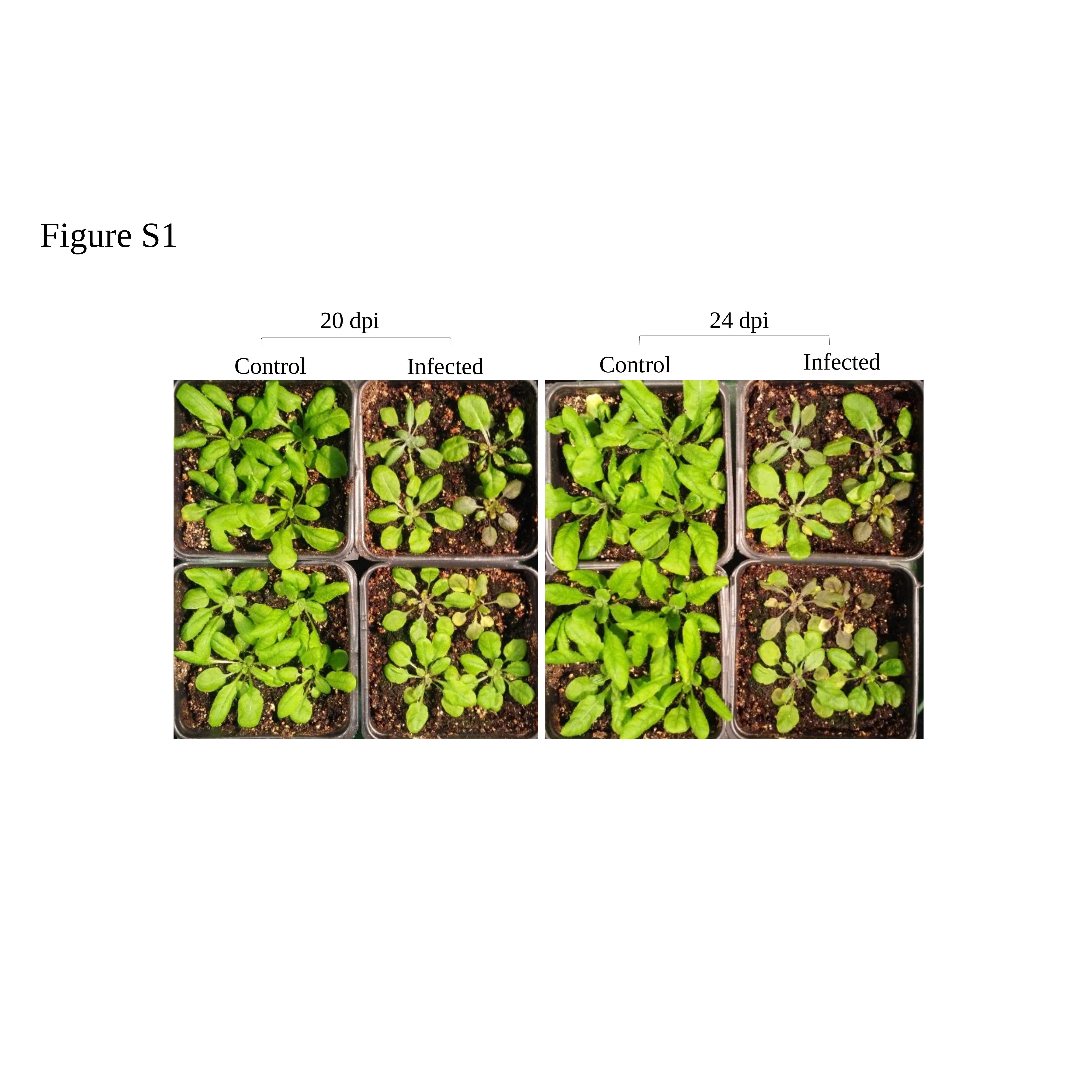

Figure S1
24 dpi
20 dpi
Infected
Control
Control
Infected

## Slide 2
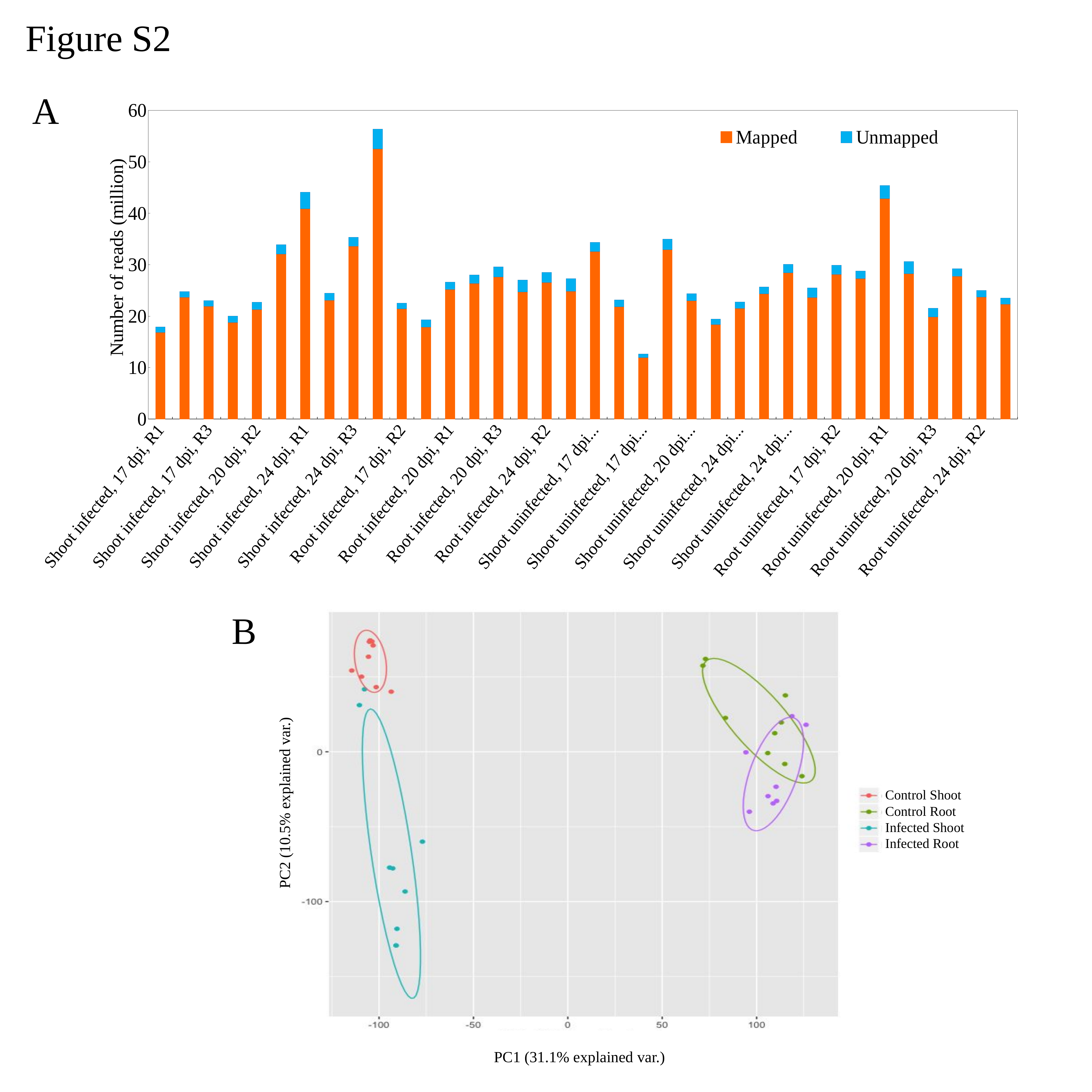

Figure S2
A
### Chart
| Category | Mapped | Unmapped |
|---|---|---|
| Shoot infected, 17 dpi, R1 | 16.850948 | 1.047192 |
| Shoot infected, 17 dpi, R2 | 23.649494 | 1.142868 |
| Shoot infected, 17 dpi, R3 | 21.92064999999999 | 1.098686 |
| Shoot infected, 20 dpi, R1 | 18.754484 | 1.283898 |
| Shoot infected, 20 dpi, R2 | 21.2937 | 1.428362 |
| Shoot infected, 20 dpi, R3 | 32.067826 | 1.855722 |
| Shoot infected, 24 dpi, R1 | 40.867262 | 3.229346 |
| Shoot infected, 24 dpi, R2 | 23.100694 | 1.37477 |
| Shoot infected, 24 dpi, R3 | 33.59742600000001 | 1.740674 |
| Root infected, 17 dpi, R1 | 52.491686 | 3.856009999999999 |
| Root infected, 17 dpi, R2 | 21.40935 | 1.121076 |
| Root infected, 17 dpi, R3 | 17.84841399999999 | 1.439144 |
| Root infected, 20 dpi, R1 | 25.19575 | 1.433612 |
| Root infected, 20 dpi, R2 | 26.35546799999999 | 1.677378 |
| Root infected, 20 dpi, R3 | 27.605076 | 1.990196 |
| Root infected, 24 dpi, R1 | 24.689084 | 2.326916 |
| Root infected, 24 dpi, R2 | 26.530888 | 1.994892 |
| Root infected, 24 dpi, R3 | 24.82456000000001 | 2.488284 |
| Shoot uninfected, 17 dpi, R1 | 32.544374 | 1.791394 |
| Shoot uninfected, 17 dpi, R2 | 21.804368 | 1.379822 |
| Shoot uninfected, 17 dpi, R3 | 11.92973 | 0.705162 |
| Shoot uninfected, 20 dpi, R1 | 32.9047 | 2.059553999999999 |
| Shoot uninfected, 20 dpi, R2 | 23.010956 | 1.378686 |
| Shoot uninfected, 20 dpi, R3 | 18.36473 | 1.057908 |
| Shoot uninfected, 24 dpi, R1 | 21.501228 | 1.267412 |
| Shoot uninfected, 24 dpi, R2 | 24.31581600000001 | 1.36995 |
| Shoot uninfected, 24 dpi, R3 | 28.42616 | 1.67317 |
| Root uninfected, 17 dpi, R1 | 23.62511599999999 | 1.87095 |
| Root uninfected, 17 dpi, R2 | 28.130916 | 1.778006 |
| Root uninfected, 17 dpi, R3 | 27.29966 | 1.492612 |
| Root uninfected, 20 dpi, R1 | 42.861902 | 2.534104 |
| Root uninfected, 20 dpi, R2 | 28.245684 | 2.399347999999999 |
| Root uninfected, 20 dpi, R3 | 19.857416 | 1.678908 |
| Root uninfected, 24 dpi, R1 | 27.73651 | 1.519252 |
| Root uninfected, 24 dpi, R2 | 23.713582 | 1.28749 |
| Root uninfected, 24 dpi, R3 | 22.298752 | 1.217122 |B
PC2 (10.5% explained var.)
Control Shoot
Control Root
Infected Shoot
Infected Root
PC1 (31.1% explained var.)

## Slide 3
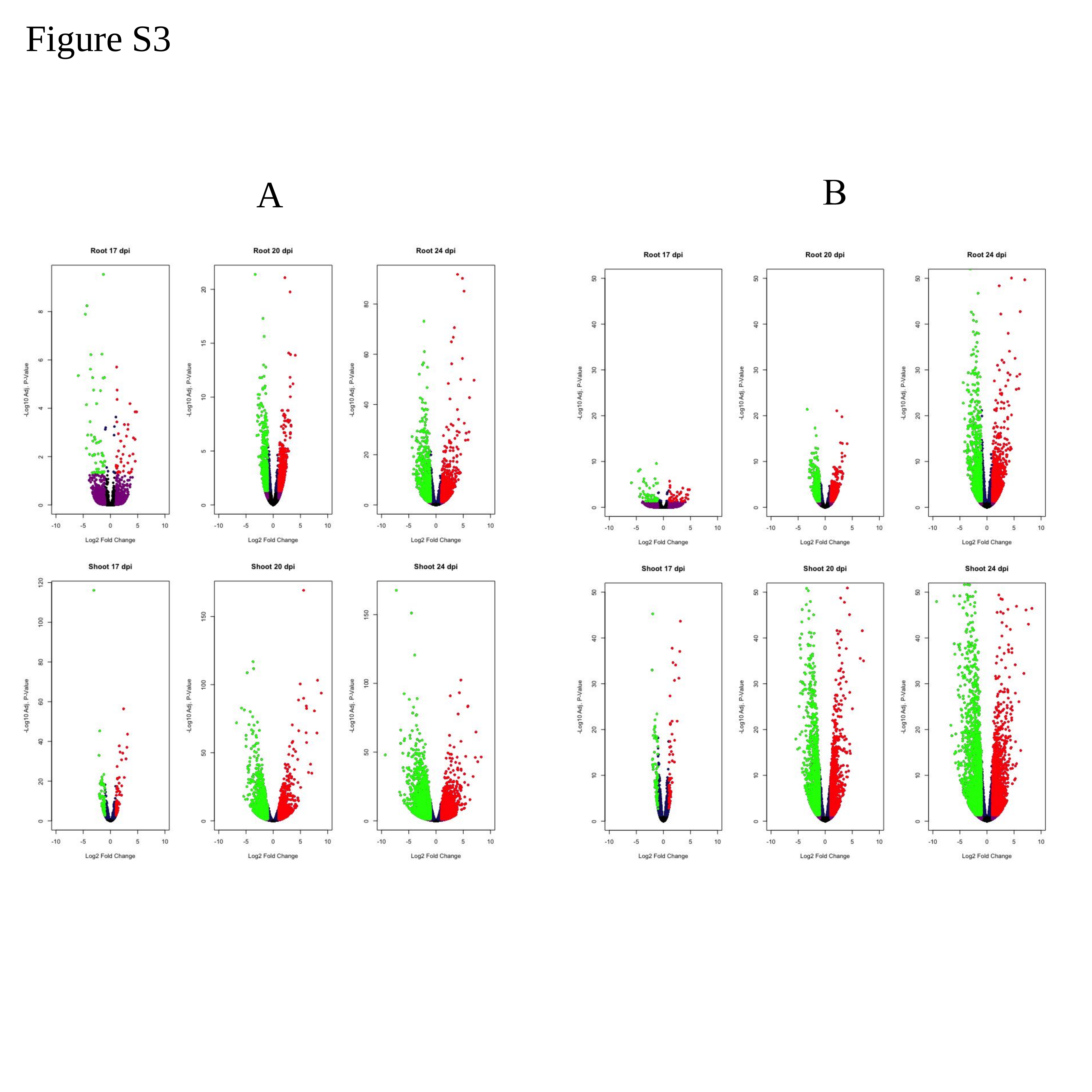

Figure S3
A

## Slide 4
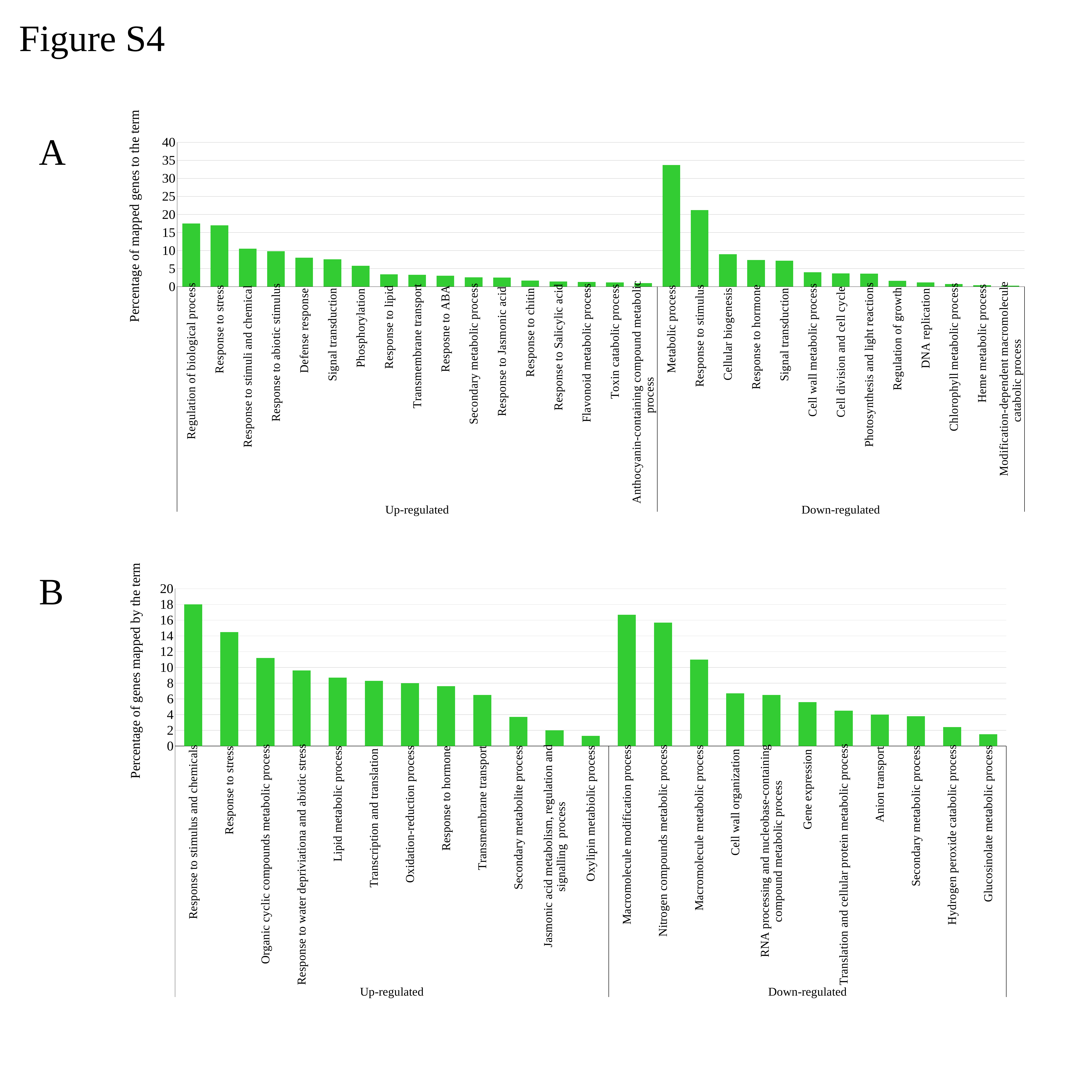

Figure S4
### Chart
| Category | |
|---|---|
| Regulation of biological process | 17.5 |
| Response to stress | 17.0 |
| Response to stimuli and chemical | 10.5 |
| Response to abiotic stimulus | 9.8 |
| Defense response | 8.0 |
| Signal transduction | 7.6 |
| Phosphorylation | 5.8 |
| Response to lipid | 3.4 |
| Transmembrane transport | 3.3 |
| Resposne to ABA | 3.0 |
| Secondary metabolic process | 2.6 |
| Response to Jasmonic acid | 2.5 |
| Response to chitin | 1.7 |
| Response to Salicylic acid | 1.4 |
| Flavonoid metabolic process | 1.3 |
| Toxin catabolic process | 1.2 |
| Anthocyanin-containing compound metabolic process | 1.0 |
| Metabolic process | 33.7 |
| Response to stimulus | 21.2 |
| Cellular biogenesis | 9.0 |
| Response to hormone | 7.4 |
| Signal transduction | 7.2 |
| Cell wall metabolic process | 4.0 |
| Cell division and cell cycle | 3.7 |
| Photosynthesis and light reactions | 3.6 |
| Regulation of growth | 1.6 |
| DNA replication | 1.2 |
| Chlorophyll metabolic process | 0.7 |
| Heme metabolic process | 0.4 |
| Modification-dependent macromolecule catabolic process | 0.3 |A
### Chart
| Category | |
|---|---|
| Response to stimulus and chemicals | 18.0 |
| Response to stress | 14.5 |
| Organic cyclic compounds metabolic process | 11.2 |
| Response to water depriviationa and abiotic stress | 9.6 |
| Lipid metabolic process | 8.700000000000001 |
| Transcription and translation | 8.3 |
| Oxidation-reduction process | 8.0 |
| Response to hormone | 7.6 |
| Transmembrane transport | 6.5 |
| Secondary metabolite process | 3.7 |
| Jasmonic acid metabolism, regulation and signalling process | 2.0 |
| Oxylipin metabiolic process | 1.3 |
| Macromolecule modification process | 16.7 |
| Nitrogen compounds metabolic process | 15.7 |
| Macromolecule metabolic process | 11.0 |
| Cell wall organization | 6.7 |
| RNA processing and nucleobase-containing compound metabolic process | 6.5 |
| Gene expression | 5.6 |
| Translation and cellular protein metabolic process | 4.5 |
| Anion transport | 4.0 |
| Secondary metabolic process | 3.8 |
| Hydrogen peroxide catabolic process | 2.4 |
| Glucosinolate metabolic process | 1.5 |B

## Slide 5
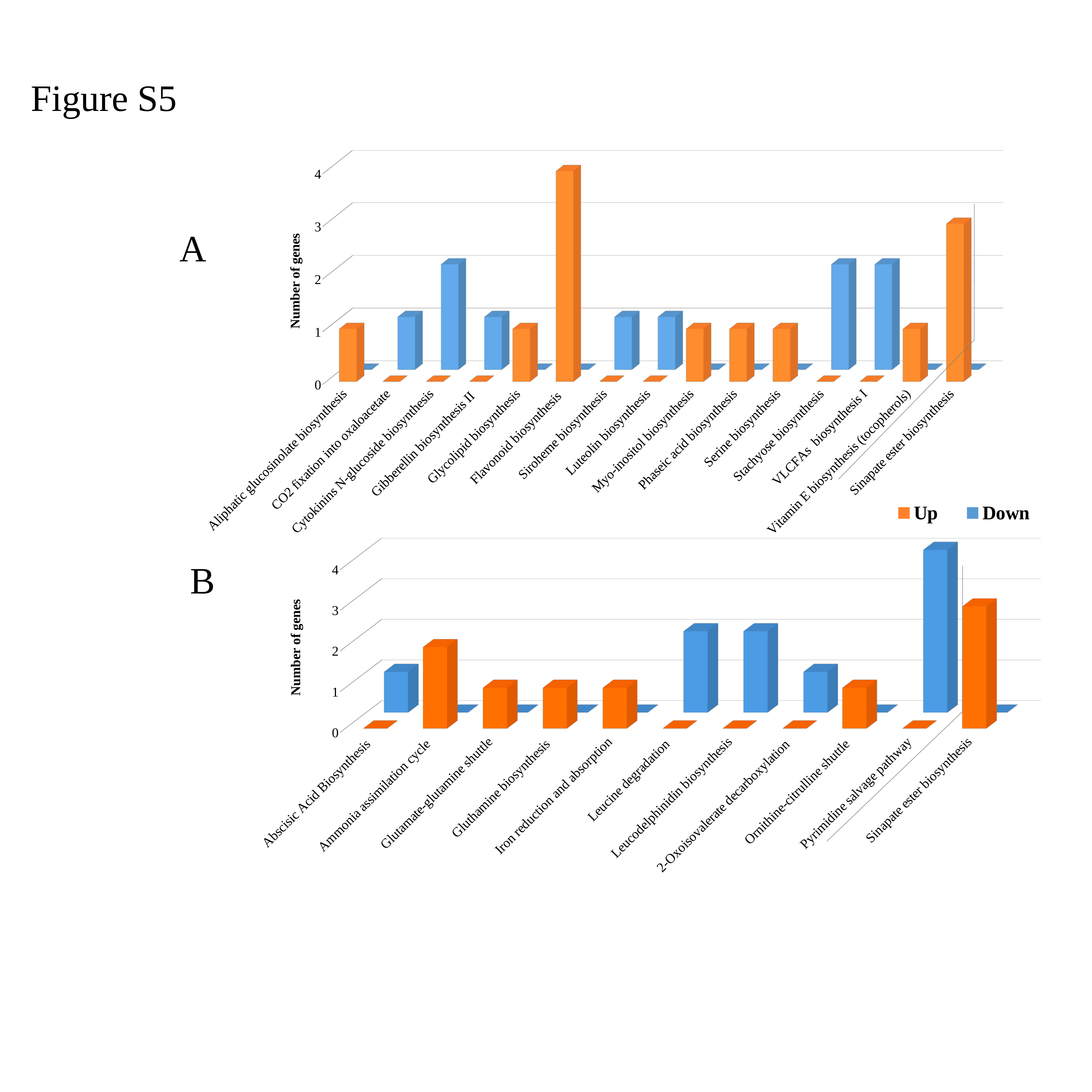

Figure S5
[unsupported chart]
A
[unsupported chart]
B

## Slide 6
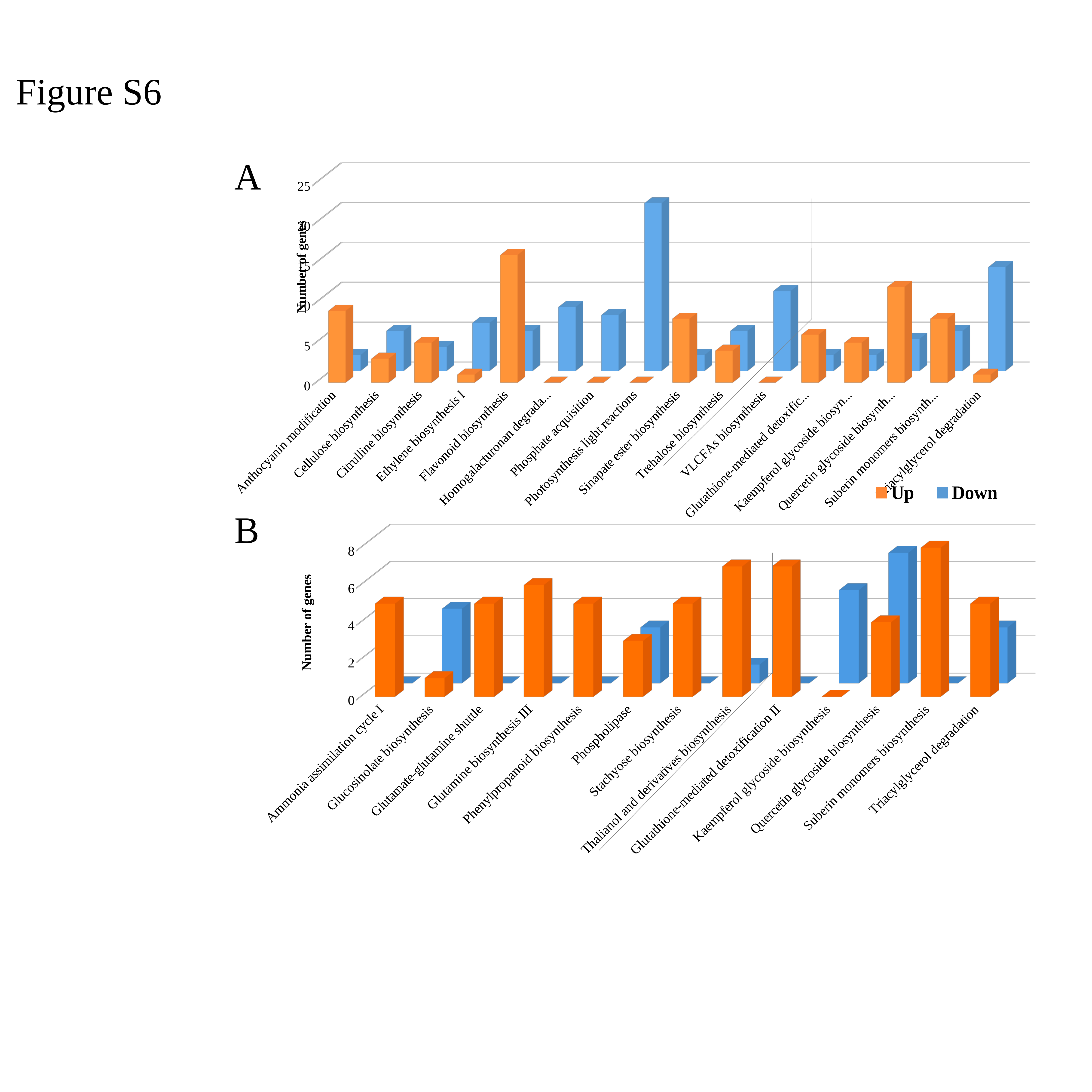

Figure S6
[unsupported chart]
A
[unsupported chart]
B
